# Interdevice Agreement of Keratometry, Astigmatism Vectors, and Ocular Biometry in Cataract Candidates: SS-OCT (Argos) vs. OLCI (Aladdin) vs. Scheimpflug–Placido (Sirius)

**DOI:** 10.3390/bioengineering13030296

**Published:** 2026-03-03

**Authors:** Leila Al Barri, Ionela-Iasmina Yasar, Nadina Mercea, Anca Tudor, Horia T. Stanca, Cosmin Roșca, Mihnea Munteanu

**Affiliations:** 1 Ophthalmology Department, “Victor Babes” University of Medicine and Pharmacy, 300041 Timisoara, Romania; leila.albarri@umft.ro (L.A.B.); ionela.yasar@umft.ro (I.-I.Y.); nadina.mercea@umft.ro (N.M.); mihnea.munteanu@umft.ro (M.M.); 2 Medical Informatics and Biostatistics Department, “Victor Babes” University of Medicine and Pharmacy, 300041 Timisoara, Romania; atudor@umft.ro; 3 Ophthalmology Department, “Carol Davila” University of Medicine and Pharmacy, 050474 Bucharest, Romania; horia.stanca@umfcd.ro; 4 Oculens Clinic, Calea Turzii No. 134-136, 400347 Cluj Napoca, Romania

**Keywords:** corneal topography, cataract surgery, intraocular lens (IOL), biometry, swept-source optical coherence tomography (SS-OCT), optical low-coherence interferometry (OLCI)

## Abstract

**Background and Objectives:** Accurate anterior segment measurements are central to intraocular lens (IOL) power calculation and toric planning, yet different optical platforms may yield non-interchangeable values. This study compared keratometry, astigmatism metrics, and ocular biometry obtained with a swept-source OCT biometer (Argos), an optical low-coherence interferometry biometer (Aladdin), and a combined Scheimpflug–Placido topographer (Schwind Sirius). **Methods:** This is a retrospective observational study (January 2022–June 2024) including eyes undergoing uncomplicated cataract surgery. All eyes were measured in a single session by one examiner. Outcomes included K1, K2, cylinder, astigmatism axis (degrees; device-reported corneal cylinder axis, labeled “Powerful Angle” in the Sirius export), vector components (J0 and J45), and—where available—lens thickness (LT), axial length (AL), anterior chamber depth (ACD), white-to-white (WTW) distance, and central corneal thickness (CCT). Friedman tests assessed 3-device differences, and pairwise comparisons were evaluated using Wilcoxon signed-rank tests (paired data). **Results:** A total of 170 eyes (102 patients) were analyzed (mean age: 69.12 ± 10.26 years). Significant inter-device differences were detected for K1 (Argos: 43.45 ± 1.64 D; Aladdin: 43.41 ± 1.70 D; overall: *p* < 0.001; Argos vs. Aladdin: *p* = 0.019), K2 (Argos: 44.45 ± 1.67 D; Aladdin: 44.34 ± 1.71 D; overall and pairwise: *p* < 0.001), and cylinder (Argos: −0.83 ± 0.74 D, Aladdin: −0.77 ± 0.76 D; Sirius: −0.68 ± 0.75 D; overall: *p* < 0.001). “Powerful Angle” differed across devices (*p* = 0.003) but not between Argos and Aladdin (*p* = 0.512). J0 (*p* = 0.277) and J45 (*p* = 0.084) did not differ significantly. Argos reported higher ACD (3.19 ± 0.42 vs. 3.13 ± 0.41 mm, *p* < 0.001) and WTW (11.95 ± 0.42 vs. 11.65 ± 0.39 mm, *p* < 0.001) values than Aladdin. CCT was similar between Aladdin and Sirius (540.27 ± 33.44 vs. 540.47 ± 33.78 µm, *p* = 0.169). **Conclusions:** Several keratometric and biometric parameters differed significantly by device, indicating limited interchangeability—particularly relevant for toric and premium IOL planning—while vector astigmatism components and CCT showed better agreement.

## 1. Introduction

The precise measurement of anterior segment parameters is paramount in contemporary cataract surgery, influencing the accuracy of intraocular lens power calculation, the final refractive outcome and the diagnosis and management of various corneal pathologies [1].

A multitude of technologies have emerged to facilitate these measurements, each with its own set of advantages and limitations. Among these technologies, swept-source optical coherence tomography (SS-OCT) biometers have gained prominence due to their high-speed scanning capabilities and enhanced penetration through dense media, enabling comprehensive anterior segment imaging [2]. The Argos biometer (MOVU Inc., Komaki, Japan), which employs SS-OCT technology, features a light source centered at 1050 nm. The narrow-bandwidth wavelength of this light source enhances the signal-to-noise ratio, enabling improved tissue penetration and image quality, even in the presence of dense cataracts [3].

Optical low-coherence interferometry (OLCI) biometers, such as Aladdin (Topcon, Tokyo, Japan), represent an older generation of optical biometers capable of non-invasively measuring axial length (AL), anterior chamber depth (ACD), pupillometry, corneal keratometry (K), and white-to-white topography (WTW). This biometer employs a 24-ring Placido disk reflection, with an approximate working distance of eight centimeters, to perform a precise keratometry and corneal topography assessment. However, the accuracy of measurements obtained from an OLCI biometer may be impacted by the presence of media opacities, such as hard cataracts [4].

Corneal topographers employing combined Scheimpflug–Placido disk technology like Schwind Sirius (SCHWIND eye-tech-solutions, GmbH, Kleinostheim) provide detailed information about corneal curvature, elevation, and thickness, which is essential for refractive surgery planning and the diagnosis of corneal ectasia. These devices not only provide valuable data that can be used for planning laser-assisted in situ keratomileusis (LASIK) treatments but also choose a toric intraocular lens, correcting astigmatism and optimizing visual outcomes after cataract surgery and refractive procedures [5].

Persistent astigmatism post-cataract intervention can significantly impair visual quality and patient satisfaction. Approximately 40% of individuals undergoing cataract surgery exhibit corneal astigmatism exceeding 1 D, which can be addressed using a toric intraocular lens [6]. As lenticular astigmatism is eliminated during cataract surgery, the measurement of total corneal astigmatism (anterior and posterior) remains critical in surgical planning. Measuring anterior corneal astigmatism requires combining various methods, such as keratometry, topography, Scheimpflug imaging, and OCT, to account for irregular astigmatism or ectatic disease that may not be apparent with a single technique. Accurately measuring posterior corneal astigmatism poses challenges, but techniques such as Scheimpflug imaging, OCT, and light-emitting diode-based devices, used in conjunction with theoretical formulas like the Barrett toric calculator, can help to compensate for this [7]. More broadly, rapid innovation in ophthalmology—including emerging therapeutic delivery approaches and biomarker-driven ocular disease profiling—reinforces the need for standardized, high-fidelity ocular measurements across technologies, particularly when measurements inform clinical decision-making and device-dependent calculations [8].

Given the need for precision, this study aims to compare the measurements obtained across different devices in order to determine the degree of agreement between them and to identify any potential sources of variability.

## 2. Materials and Methods

### 2.1. Study Design, Ethics, and Informed Consent

This retrospective, observational study, conducted at the Ophthalmology Department of the “Victor Babes” University of Medicine and Pharmacy in Timisoara, Romania, from January 2022 to June 2024, evaluated the measurements done to patients undergoing successful cataract surgery. The study adhered to the principles of the Declaration of Helsinki, and the institutional ethics committee waived specific approval requirements due to its retrospective nature. Furthermore, the informed consent obtained from all participants for their surgical procedures explicitly included consent for the use of their data in research studies. Importantly, patient confidentiality was maintained throughout the study.

### 2.2. Patients and Procedure

Subjects included were those undergoing uneventful cataract surgery with intraocular lens (IOL) implantation. The exclusion criteria included ocular trauma, severe corneal or vitreous opacities, previous corneal surgery, retinal disease, and systemic disease affecting the eye. All patients were measured with the three devices in a single session by the same examiner and faced no difficulties. The following parameters were obtained and compared: K1, K2 and cylinder on all devices. Corneal astigmatism is represented as vector components J0 and J45, calculated according to the following formulas [8]:

○ J0 = −(C/2) * cos(2*axis).
○ J45 = −(C/2) * sin(2*axis).
○ *(C = cylinder or K2* − *K1, A = axis)*.

Central corneal thickness (CCT) was measured and compared between Aladdin and Schwind Sirius.

Additionally, white-to-white (WTW) distance, lens thickness (LT) and anterior chamber depth (ACD) were measured using the two biometers SS-OCT Argos and OLCI Aladdin. All devices were calibrated prior to the measurement session.

Some device exports label the corneal astigmatism axis as “Powerful Angle”. In this manuscript, this parameter is reported as the astigmatism axis (degrees) and refers to the axis of the device-reported anterior corneal cylinder/steep meridian, expressed on a 0–180° scale.

All devices underwent manufacturer-recommended internal calibration/self-check procedures prior to measurement sessions. The instruments were maintained under routine preventive maintenance/service schedules by the suppliers.

All measurements were obtained in a single session by one trained examiner; therefore, inter-observer variability was not assessed in this study.

### 2.3. Devices

The Argos SS-OCT device generates 2D OCT images at 3000 A-scans/s using a 1060 nm light. It measures corneal curvature using a 1.3375 refractive index and a 2.2 mm ring of 16 LEDs combined with OCT data. The corneal diameter is also determined from an OCT image, and the newer Argos 1.5 version uses this to calculate the white-to-white value. Additionally, the device measures central corneal thickness (CCT), anterior chamber depth (ACD), lens thickness (LT), and axial length (AL) using OCT data with varied refractive indices (cornea: 1.376; aqueous and vitreous humors: 1.336; lens: 1.410). However, CCT measurement is not readily available on the report, unlike other parameters.

Argos derives keratometry from OCT information combined with a small, ~2.2 mm, LED ring sample; Aladdin computes keratometry from Placido-based reflections across an approximately ~3.0 mm zone (2.8–3.2 mm); and Sirius provides simulated keratometry derived from Placido/Scheimpflug-based anterior surface reconstruction (commonly reported as SimK around the central, ~3 mm). These differences in sampling geometry and reconstruction are expected to contribute to systematic differences across devices. Although Argos can derive CCT from OCT, in our routine reporting/export configuration CCT was not consistently available as a structured variable across the full retrospective period; therefore, CCT agreement was evaluated between Aladdin and Sirius where CCT was consistently captured.

The Aladdin biometer is based on OLCI, with an 820 mm superluminescent diode, and is used for measuring AL, ACD, CCT, and LT. Keratometry (K) readings are calculated using a 24-ring Placido disk reflection, which captures data from approximately 1024 reference points within a 3.0 mm (from 2.8 to 3.2 mm) optical zone on the corneal surface. The WTW distance is calculated from the corneal topography.

The Schwind Sirius topography instrument is an anterior segment analysis system that combines a rotating Scheimpflug camera and a Placido disk. It acquires 25 radial sections of the cornea and anterior chamber. The system provides data on the tangential and axial curvature of the anterior and posterior corneal surfaces, the overall refractive power of the cornea, corneal thickness mapping, and wavefront analysis. The corneal surfaces are examined using blue LED light. Measurements of the anterior corneal surface are obtained by combining Placido and Scheimpflug data, while measurements of other internal structures rely solely on Scheimpflug imaging.

### 2.4. Statistical Analysis

As a retrospective study, the sample size was defined by the number of eligible eyes with complete same-session measurements across devices. To contextualize adequacy, we performed a post hoc power calculation using a clinically relevant keratometry difference of 0.25 D and the observed dispersion of paired inter-device differences. With *n* = 170 eyes, the dataset provides >90% power to detect a 0.25 D paired difference at α = 0.05.

Because the astigmatism axis is circular (0–180°), linear mean/SD can be misleading. The axis is therefore summarized using median (IQR) and/or circular descriptive statistics, while inferential comparisons emphasize the vector representation J0 and J45, which avoids axis wrap-around artifacts.

Data analysis was performed with JASP v0.19.3 (open-source software for statistical analysis, supported by the University of Amsterdam). Mean and standard deviation, median and interquartile range were used to present numerical variables, while frequency and percentages were used for nominal variables. The distribution of continuous variables was assessed using the Shapiro–Wilk Test for normality of numerical data distribution. For a non-Gaussian distribution of the data, we compared two groups using Wilcoxon signed-rank tests, and more than two groups were compared using the non-parametric Friedman Test. A *p*-value of less than 0.05 was considered statistically significant.

Because some participants contributed both eyes, primary analyses were complemented by patient-level sensitivity analyses to mitigate inter-eye correlation. Specifically, we repeated key comparisons using one randomly selected eye per patient and additionally confirmed inferences using a patient-clustered framework (mixed-effects models with patient-level random intercepts or cluster-robust standard errors). Results were considered robust when findings were concordant across approaches.

To complement hypothesis testing, we computed the root-mean-square (RMS) difference between device pairs for key variables (K1, K2, cylinder, ACD, and WTW). We also explored associations between RMS disagreement and age using Spearman’s correlation (and/or regression) to assess whether disagreement increases in older participants.

## 3. Results

In this study, a total of 170 eyes from 102 patients with uncomplicated cataracts were evaluated. The mean age of the participants was 69.12 ± 10.26 years (ranging from 40 to 86 years). All eyes were successfully measured using the three devices, and no issues arose during the process (Table 1). Consistent with the circular nature of axis data, interpretation focused primarily on vector components (J0/J45) rather than linear dispersion of axis values. Findings were robust in sensitivity analyses restricted to one eye per patient and in patient-clustered analyses, indicating that statistical significance was not solely driven by inclusion of both eyes. RMS differences between devices provide an estimate of typical disagreement magnitude beyond statistical significance.

**Figure 1:**
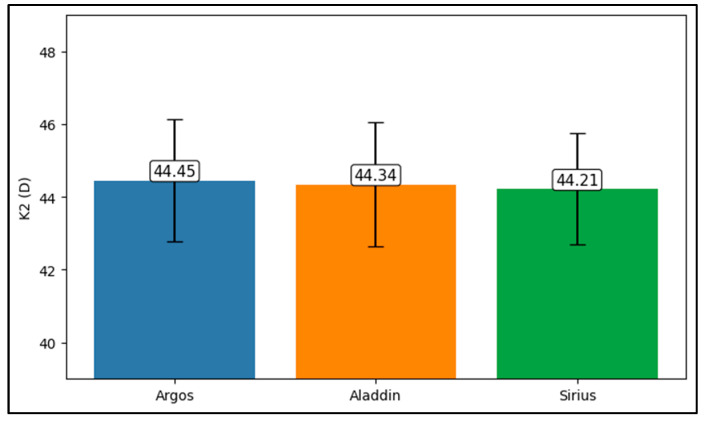
Flat keratometry (K1) measurements across Argos SS-OCT, Aladdin OLCI, and Schwind Sirius (Friedman test).

**Figure 2:**
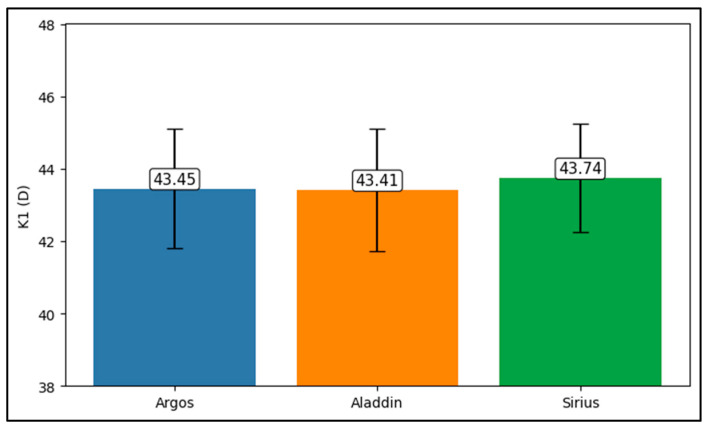
Steep keratometry (K2) measurements across Argos SS-OCT, Aladdin OLCI, and Schwind Sirius (Friedman test).

**Figure 3:**
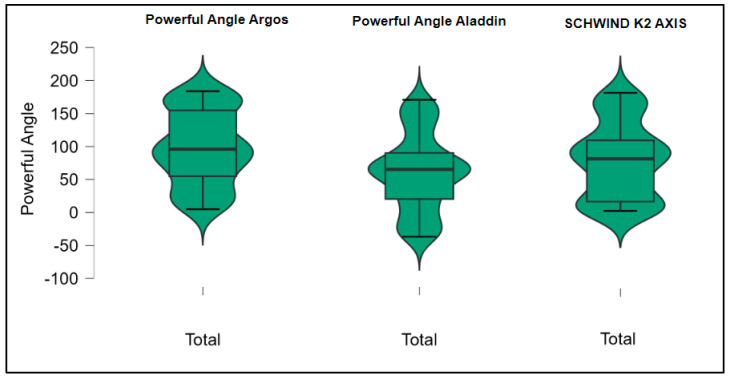
Astigmatism axis measurements across Argos SS-OCT, Aladdin OLCI, and Schwind Sirius (Friedman test).

**Figure 4:**
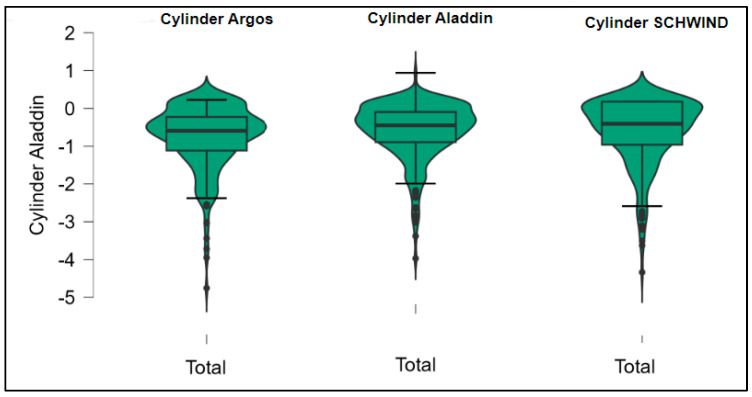
Corneal cylinder measurements across Argos SS-OCT, Aladdin OLCI, and Schwind Sirius (Friedman test).

Further analysis revealed significant disparities in several key parameters across the devices, particularly in keratometry readings, cylinder, lens thickness, axial length, anterior chamber depth, and white-to-white measurements (*p* < 0.001 for all, except for J0 and J45 components). Conversely, measurements of J0, J45, and central corneal thickness showed no statistically significant differences between the devices, suggesting a higher level of agreement for these specific parameters. Figure 1, Figure 2, Figure 3 and Figure 4 show the differences in K1, K2, astigmatism axis (degrees) and cylinder measurements across the three devices (Friedman test). In the case of these variables, further comparisons of measurements between two devices were conducted (Mann–Whitney U test).

**Figure 5:**
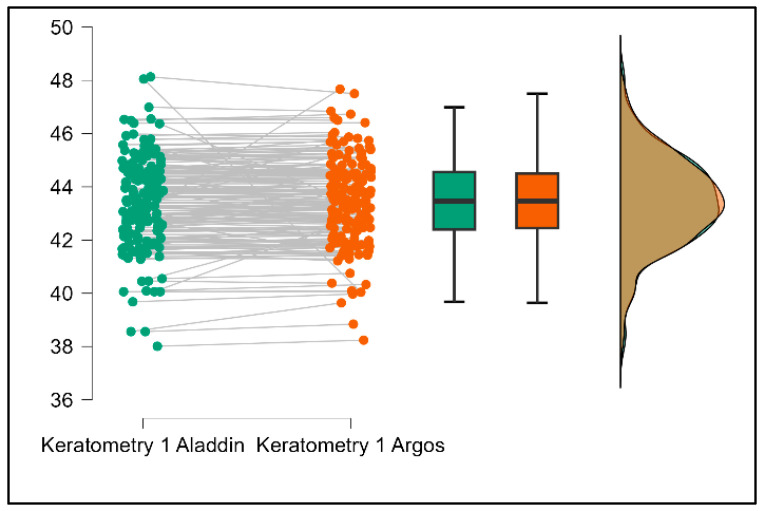
Pairwise comparison of flat keratometry (K1) between Argos SS-OCT and Aladdin OLCI biometers (Wilcoxon signed-rank test).

**Figure 6:**
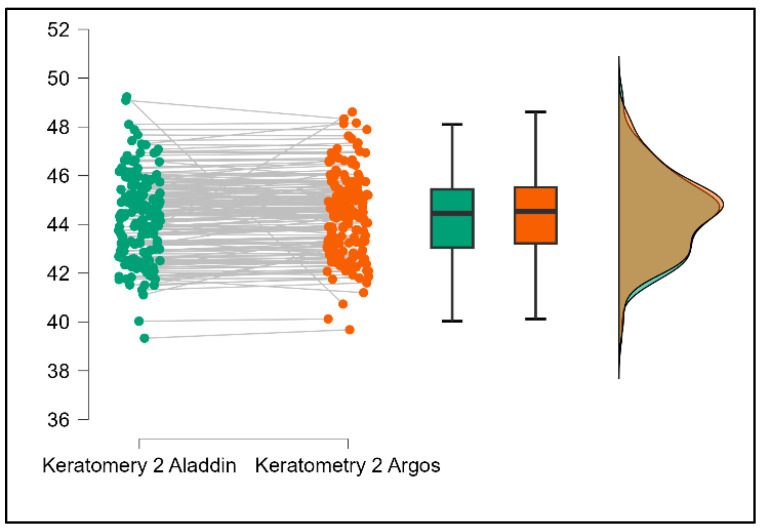
Pairwise comparison of steep keratometry (K2) between Argos SS-OCT and Aladdin OLCI biometers (Wilcoxon signed-rank test).

**Figure 7:**
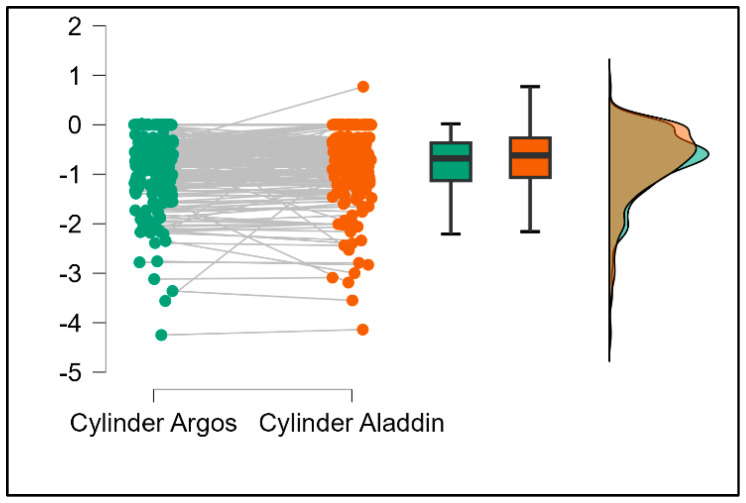
Pairwise comparison of corneal cylinder between Argos SS-OCT and Aladdin OLCI biometers (Wilcoxon signed-rank test).

**Figure 8:**
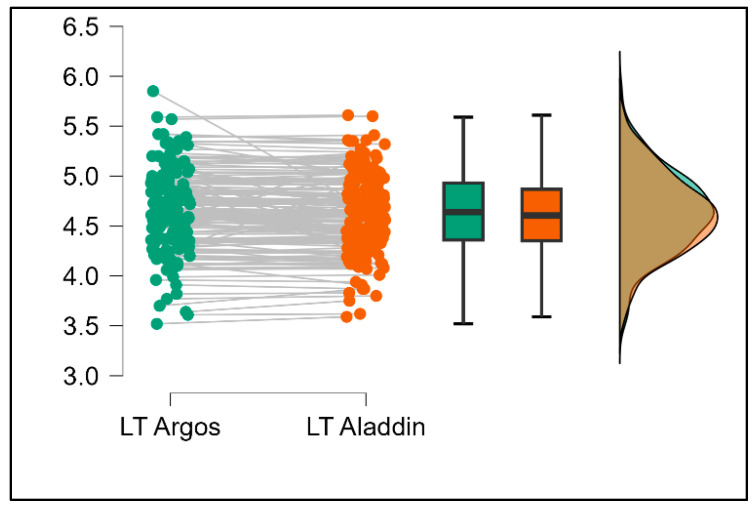
Pairwise comparison of lens thickness (LT) between Argos SS-OCT and Aladdin OLCI biometers (Wilcoxon signed-rank test).

**Figure 9:**
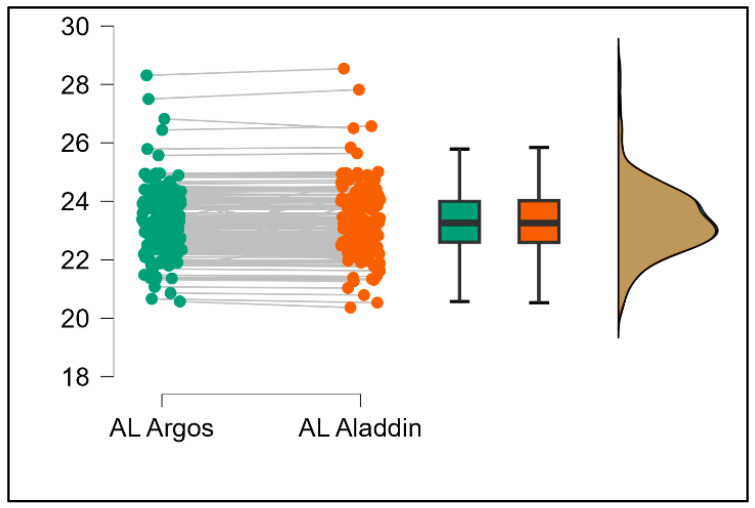
Pairwise comparison of axial length (AL) between Argos SS-OCT and Aladdin OLCI biometers (Wilcoxon signed-rank test).

**Figure 10:**
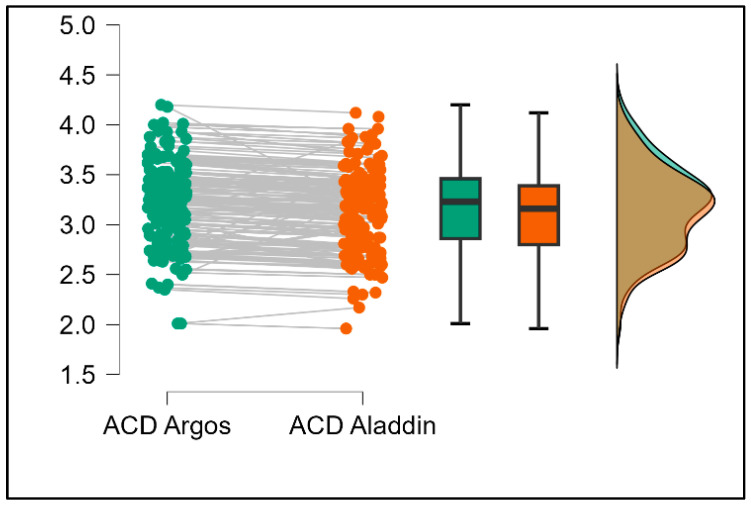
Pairwise comparison of anterior chamber depth (ACD) between Argos SS-OCT and Aladdin OLCI biometers (Wilcoxon signed-rank test).

**Figure 11:**
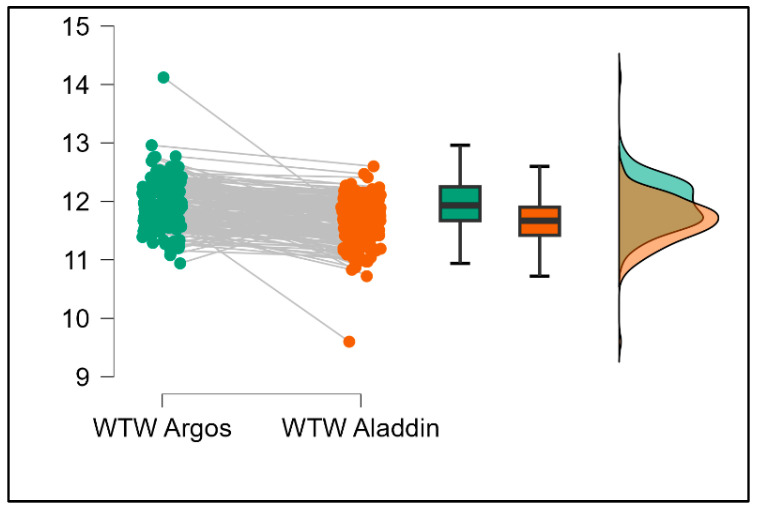
Pairwise comparison of white-to-white (WTW) distance between Argos SS-OCT and Aladdin OLCI biometers (Wilcoxon signed-rank test).

Values of K1, measured by Argos, are significantly increased compared with Aladdin (*p* = 0.019), as shown in Figure 5. Values of K2, measured by Argos, also exhibited higher values than those obtained from Aladdin (*p* < 0.001), as detailed in Figure 6. The cylinder values from Argos were significantly lower than those from Aladdin (*p* < 0.001), as shown in Figure 7. Conversely, the astigmatism axis (degrees) demonstrated no significant difference between the Argos and Aladdin devices (*p* = 0.512), indicating a consistent measurement for this parameter across the two biometers as showen in Table 2. Similarly, the Argos device consistently yielded higher measurements for lens thickness (*p* < 0.001), axial length (*p* < 0.001), anterior chamber depth (*p* < 0.001), and white-to-white distance (*p* < 0.001) when compared to Aladdin, as demonstrated in Figure 8, Figure 9, Figure 10 and Figure 11.

**Figure 12:**
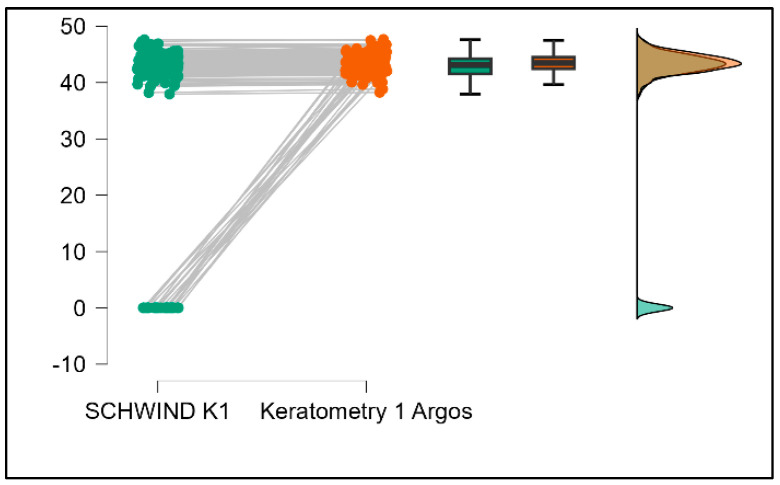
Pairwise comparison of flat keratometry (K1) between Argos SS-OCT and Schwind Sirius (Wilcoxon signed-rank test).

**Figure 13:**
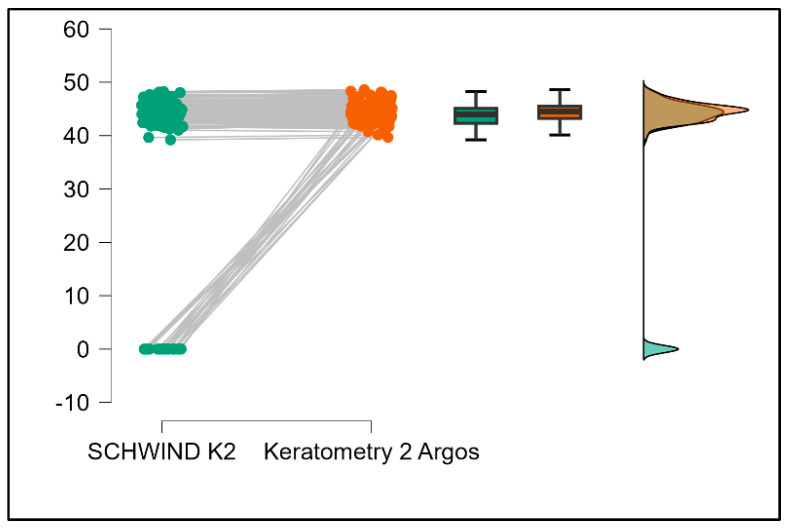
Pairwise comparison of steep keratometry (K2) between Argos SS-OCT and Schwind Sirius (Wilcoxon signed-rank test).

**Figure 14:**
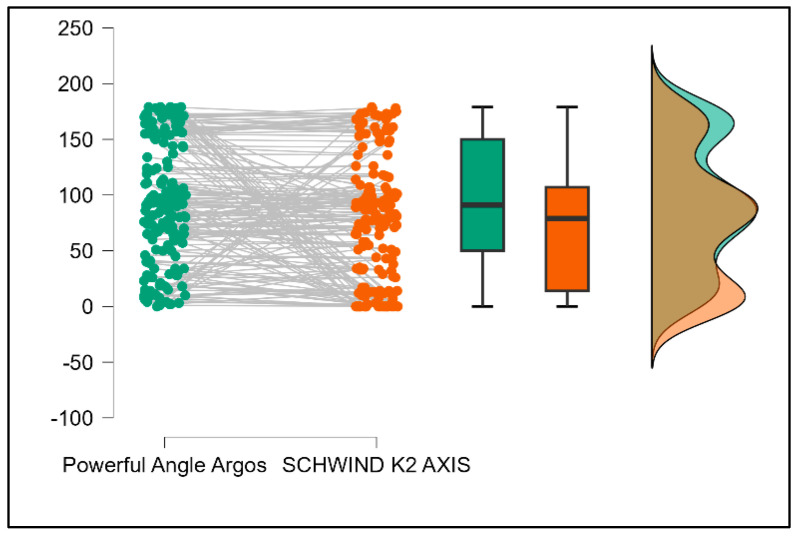
Pairwise comparison of astigmatism axis (degrees) between Argos SS-OCT and Schwind Sirius (Wilcoxon signed-rank test).

**Figure 15:**
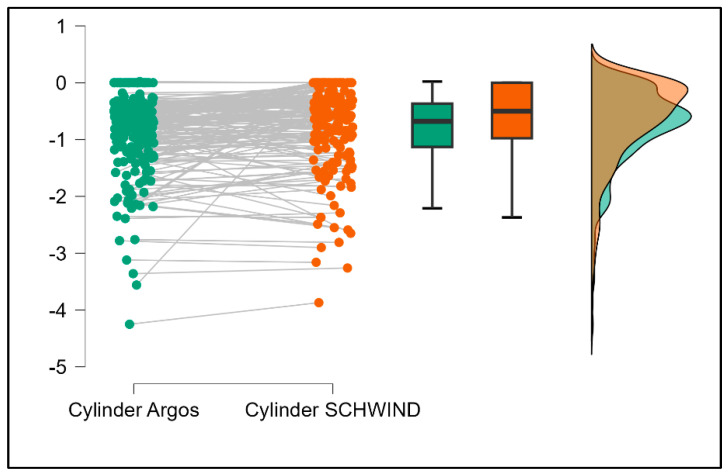
Pairwise comparison of corneal cylinder between Argos SS-OCT and Schwind Sirius (Wilcoxon signed-rank test).

Table 3 compares the values of keratometry readings, astigmatism axis (degrees), and cylinder between the Argos and Schwind devices. The analysis revealed that both K1 and K2 measurements from Argos were significantly higher than those from Schwind (Figure 12 and Figure 13). The average astigmatism axis (degrees) values are significantly lower in the case of Schwind compared to Argos (Mann–Whitney U test, *p* = 0.003). This pattern of difference is further accentuated by the significantly lower cylinder values observed with Argos compared to Schwind (Mann–Whitney U test, *p* < 0.001), as presented in Figure 14 and Figure 15.

**Figure 16:**
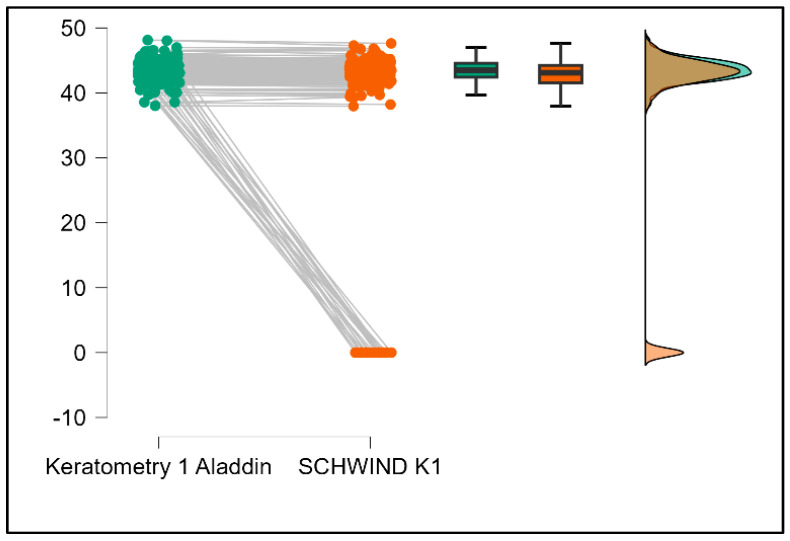
Pairwise comparison of flat keratometry (K1) between Aladdin OLCI biometer and Schwind Sirius (Wilcoxon signed-rank test).

**Figure 17:**
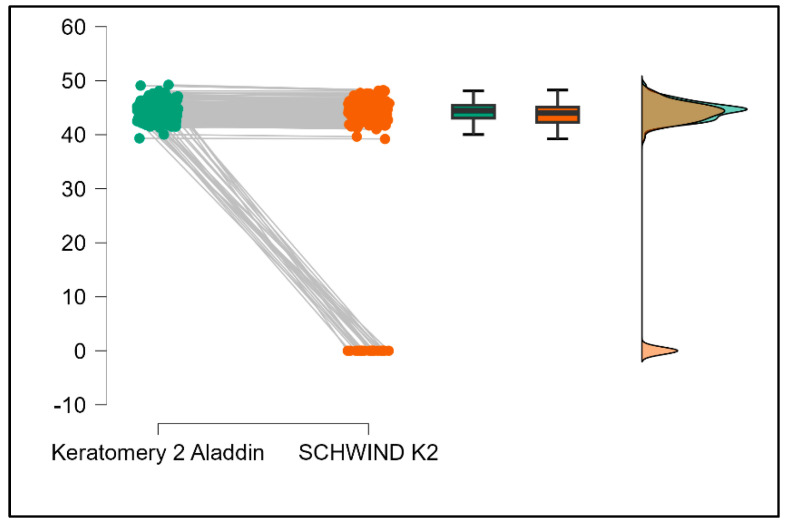
Pairwise comparison of steep keratometry (K2) between Aladdin OLCI biometer and Schwind Sirius (Wilcoxon signed-rank test).

**Figure 18:**
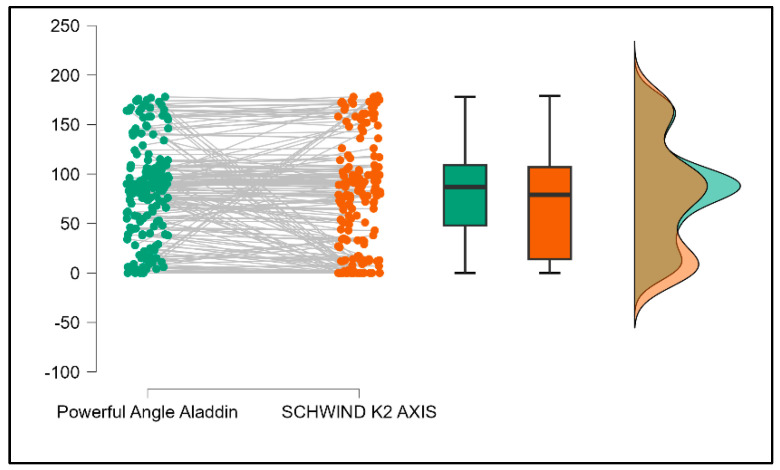
Pairwise comparison of astigmatism axis (degrees) between Aladdin OLCI biometer and Schwind Sirius (Wilcoxon signed-rank test).

**Figure 19:**
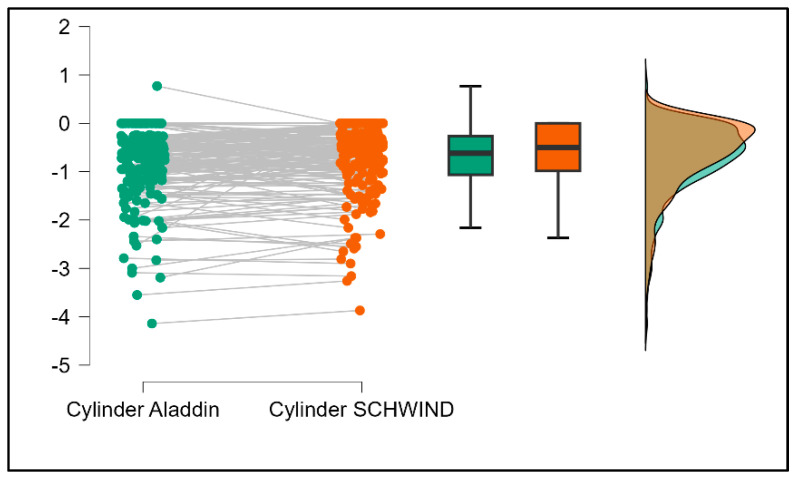
Pairwise comparison of corneal cylinder between Aladdin OLCI biometer and Schwind Sirius (Wilcoxon signed-rank test).

Table 4 shows statistically significant differences in K1, K2, astigmatism axis (degrees), and cylinder measurements when comparing the Aladdin and Schwind devices, with Aladdin consistently yielding higher values across these parameters (Figure 16, Figure 17, Figure 18 and Figure 19). The average astigmatism axis (degrees) values are significantly lower in the case of Schwind compared to Alladin (Mann–Whitney U test, *p* = 0.009), and the average cylinder values are significantly lower in the case of Schwind compared to Aladdin (Mann–Whitney U test, *p* = 0.002).

## 4. Discussion

In cataract surgery, the precise measurement of anterior segment parameters in conjunction with axial length is essential for accurate intraocular lens power calculation. Inaccurate measurements of axial length, keratometry, and anterior chamber depth have been identified as the primary sources of errors in intraocular lens power calculations. Errors in predicted postoperative ACD/effective lens position are a major contributor to refractive surprise, and even relatively small position errors can translate into clinically meaningful refractive errors, with the magnitude depending on ocular geometry and IOL power [9]. Importantly, recent comparative analyses of modern IOL power calculation formulas have shown that, despite advances in formula design, the accuracy of postoperative refractive outcomes remains highly dependent on the precision of the underlying biometric measurements, and when using a toric intraocular lens, even minor inaccuracies in keratometric axis measurements can lead to incorrect axis orientation and suboptimal astigmatic correction [10]. Given the critical importance of these parameters, evaluating the agreement between different biometry devices is essential for ensuring interchangeability and optimizing surgical planning, particularly in the context of advancing lens technologies and increasing patient expectations for precise refractive outcomes [11].

In this study we assessed the agreement and interchangeability of biometric measurements obtained from three different machines, two optical biometers—Argos and Aladdin—and one Scheimpflug–Placido disk corneal topographer—Schwind Sirius. Our findings indicate statistically significant differences in various ocular parameters across these devices, challenging their direct interchangeability in some clinical contexts.

The statistically significant differences observed in keratometry values (K1 and K2), astigmatism axis (degrees), cylinder, lens thickness, axial length, anterior chamber depth, and white-to-white measurements highlight the lack of interchangeability among the devices for these specific parameters, despite comparable central corneal thickness measurements [12,13]. This finding aligns with previous research indicating that while some parameters like central corneal thickness might show agreement, other anterior segment measurements and keratometric values often differ significantly between devices [12,14]. Specifically, previous studies have demonstrated that while modern biometers generally agree on axial length measurements, variations in keratometry and anterior chamber depth can lead to clinically relevant discrepancies in intraocular lens power calculations [15]. For instance, studies comparing different swept-source optical coherence tomography biometers and Scheimpflug-based devices have consistently shown strong agreement in overall parameters, yet notable discrepancies in keratometry, necessitating careful consideration when selecting IOLs [16]. Furthermore, some research has indicated that while optical biometers generally provide higher values for axial length and anterior chamber depth compared to ultrasound biometry, differences can still persist between various optical biometers for keratometry and corneal diameter [17]. These differences underscore the critical need for clinicians to be aware of the specific characteristics and potential biases of each biometer used, especially when dealing with complex cases such as post-refractive surgery eyes or those with corneal opacities [18].

The observed differences in keratometric measurements between the Argos, Aladdin, and Schwind Sirius devices can be attributed to the distinct underlying measurement principles and optical assumptions employed by each technology. The Argos SS-OCT biometer derives corneal curvature by integrating OCT-based anterior segment imaging with LED-based keratometry, whereas Aladdin relies exclusively on Placido disk-based reflection over a predefined corneal zone. In contrast, Schwind Sirius combines Placido-derived anterior surface data with Scheimpflug imaging, allowing for three-dimensional reconstruction of the cornea. These methodological differences likely account for the consistently higher keratometry values obtained with optical biometers compared to the Scheimpflug–Placido system, a finding also reported in previous comparative studies [12]. In line with our findings [19], excellent agreement between SS-OCT and OLCI biometers for axial length (ICC = 0.975), anterior chamber depth (ICC = 0.960), lens thickness (ICC = 0.951), and keratometry (K1 ICC = 0.921, K2 ICC = 0.927) has been reported, but moderate to poor agreement has been noted for astigmatism axis, the cylinder vectors J0 and J45, and white-to-white distance, emphasizing that interchangeability may be limited for parameters where precision is critical.

Despite statistically significant differences in keratometric magnitude and cylinder values, the absence of significant differences in the vector components J0 and J45 suggests a relatively preserved agreement in astigmatic orientation across devices. This finding is clinically relevant, as vector analysis has been shown to better represent astigmatic agreement than conventional cylinder and axis values alone [8]. In addition, comparative analysis of refractive predictive accuracy showed that both SS-OCT and OLCI technologies deliver high refractive predictability in clinical practice, with more than 97% of eyes achieving postoperative spherical equivalent outcomes within ±1.00 D of target refraction and excellent uncorrected distance visual acuity regardless of biometer type, although subtle differences in lens thickness and anterior chamber depth prediction were observed [20]. From a surgical planning perspective, this implies that while absolute keratometric values may not be interchangeable, the directional consistency of astigmatism remains largely comparable among devices, which may partially mitigate the clinical impact of measurement discrepancies when selecting toric intraocular lenses.

The relative agreement in J0 and J45 despite statistically significant differences in scalar cylinders is plausible for several reasons. First, vector decomposition expresses astigmatism as orthogonal components, reducing sensitivity to the 0/180° wrap-around problem that can distort arithmetic means of axis values. Second, devices may differ in sampling zone, smoothing, and keratometric index assumptions, which can shift the magnitude of cylinders while preserving the underlying directional structure of astigmatism for most regular corneas. Clinically, this pattern suggests that toric IOL axis planning may be less affected when vector orientation is consistent, but toric power selection may still vary because even small systematic differences in keratometric magnitude translate into different cylinder estimates at the corneal plane.

Measurements of axial length, anterior chamber depth, lens thickness, and white-to-white distance were consistently higher when obtained using the Argos SS-OCT biometer compared to the Aladdin OLCI device. These findings are consistent with previously reported data demonstrating systematic offsets between SS-OCT-based and OLCI-based biometers, likely related to differences in segmentation algorithms, refractive indices, and signal penetration depths [11]. Given that modern intraocular lens power calculation formulas incorporate multiple biometric inputs, including lens thickness and anterior chamber depth, even small systematic differences may influence refractive prediction accuracy and should be considered when switching devices during preoperative assessment or postoperative follow-up [21].

Central corneal thickness measurements showed no statistically significant differences between the Aladdin and Schwind Sirius devices, suggesting good agreement for this parameter across different imaging modalities. This observation supports previous reports indicating that CCT measurements tend to be more robust and less device-dependent than keratometric or anterior chamber parameters [12]. As such, CCT values may be used interchangeably in routine clinical practice; however, caution remains warranted in cases requiring high precision, such as corneal refractive surgery or glaucoma risk assessment.

From a clinical perspective, the lack of full interchangeability among the evaluated devices highlights the importance of consistency in biometric assessment. Combining measurements obtained from different platforms may introduce cumulative errors in IOL power calculation, particularly in eyes undergoing toric or other premium IOL implantation or in patients with altered corneal anatomy [7,18]. Accordingly, clinicians should rely on a single, validated device throughout the preoperative workflow to minimize measurement-related variability and optimize refractive outcomes.

These findings support a “single-platform” approach to preoperative cataract assessment, particularly when refractive precision is critical. Although Argos and Aladdin produced similar mean keratometry values, statistically significant offsets in keratometry, cylinder, and especially anterior segment metrics used by modern formulas can compound into clinically relevant differences in lens selection, toric axis planning, and post-op refractive accuracy. Practically, clinicians should avoid mixing K/biometry inputs from different devices within the same calculation workflow; if device switching is unavoidable, consider re-deriving lens constants and repeating measurements to confirm stability, rather than assuming interchangeability.

It is important to note that the outputs of all three instruments represent device-derived estimates rather than direct physical measurements, because each platform relies on specific optical assumptions (e.g., keratometric index), segmentation boundaries, and proprietary reconstruction algorithms. Consequently, systematic offsets can occur even with correct acquisition techniques, particularly for parameters influenced by modeling choices (keratometry zone definition, WTW edge detection, or ACD boundary selection).

### Limitations

This study is limited by its retrospective, single-center design and inclusion of only uncomplicated cataract eyes, which may reduce generalizability to eyes with irregular corneas, prior refractive surgery, or advanced ocular comorbidity. Posterior corneal astigmatism and total corneal power were not directly compared across platforms. This is relevant because posterior corneal astigmatism can influence toric IOL planning, and Scheimpflug-based systems (including Sirius) can estimate posterior surface curvature. Lack of posterior-surface cross-device comparisons may therefore underestimate clinically meaningful differences in toric planning when switching platforms, particularly in eyes with non-negligible posterior astigmatism. CCT could not be compared across all three devices because Argos CCT was not consistently available in the exported dataset across the retrospective period. Although both eyes were available for some participants, we addressed potential inter-eye correlation through patient-level sensitivity and clustered analyses; residual dependence cannot be fully excluded. Repeatability/reproducibility testing (multiple repeated acquisitions per device) was not performed because the dataset reflects routine clinical measurements with a single acquisition per device. Published studies report generally good repeatability for many biometric parameters, but the lack of within-study repeatability assessment limits our ability to quantify measurement noise and device-specific precision in this cohort and should be addressed prospectively. Additionally, posterior corneal astigmatism was not directly compared across devices, an increasingly recognized contributor to total corneal power estimation. Future prospective studies incorporating postoperative refractive outcomes and agreement analyses, such as Bland–Altman plots and intraclass correlation coefficients, would further clarify the clinical relevance of the observed biometric differences.

## 5. Conclusions

In conclusion, significant differences were identified in multiple anterior segment and biometric parameters among the Argos SS-OCT biometer, Aladdin OLCI biometer, and Schwind Sirius Scheimpflug–Placido topographer. While certain parameters, such as central corneal thickness and astigmatic vector components, demonstrated acceptable agreement, others, including keratometry, axial length, anterior chamber depth, and white-to-white measurements, were not directly interchangeable. Practically, clinicians should avoid mixing keratometry/biometry inputs from different platforms within the same IOL calculation workflow. If switching devices is unavoidable, IOL power should be recalculated using device-specific lens constants (or re-optimized constants where applicable), and measurements should be verified with a repeat acquisition (and/or a second confirmatory measurement) rather than assuming interchangeability.

## Figures and Tables

**Table 1:** Descriptive statistics of keratometry, astigmatism vectors, and ocular biometry measured with Argos, Aladdin, and Schwind Sirius (Friedman test).

Variable	Argos	Aladdin	Schwind	*p*
**Keratometry 1**	43.45 ± 1.64	43.41 ± 1.7	37.4 ± 14.97	<0.001 *
	43.47 (42.45–44.5)	43.46 (42.4–44.56)	43.1 (41.52–44.23)	
**Keratometry 2**	44.45 ± 1.67	44.34 ± 1.71	38.21 ± 15.3	<0.001 *
	44.53 (43.22–45.52)	44.46 (43.05–45.44)	44 (42.3–45.1)	
**Astigmatism axis (degrees)**	92.69 ± 55.69	84.01 ± 49.35	75.98 ± 57.13	0.003 *
	91 (50–150)	87.5 (49–109)	79 (14–107)	
**Cylinder**	−0.83 ± 0.74	−0.77 ± 0.76	−0.68 ± 0.75	<0.001 *
	−0.68 (−1.13–0.37)	−0.62 (−1.07–0.27)	−0.5 (−0.98–0)	
**J0**	0.04 ± 0.35	−0.04 ± 0.42	−0.04 ± 0.37	**0.277**
	0 (−0.16–0.2)	0 (−0.21–0.13)	0 (−0.13–0.08)	
**J45**	−0.06 ± 0.42	−0.02 ± 0.38	0.05 ± 0.34	**0.084**
	0 (−0.25–0.14)	0 (−0.18–0.14)	0 (−0.09–0.18)	
**LT**	4.64 ± 0.41	4.61 ± 0.39		<0.001 *
	4.64 (4.36–4.93)	4.61 (4.35–4.87)		
**AL**	23.33 ± 1.16	23.33 ± 1.18		<0.001 *
	23.26 (22.6–24)	23.26 (22.59–24.02)		
**ACD**	3.19 ± 0.42	3.13 ± 0.41		<0.001 *
	3.23 (2.86–3.46)	3.16 (2.8–3.39)		
**WTW**	11.95 ± 0.42	11.65 ± 0.39		<0.001 *
	11.93 (11.67–12.25)	11.67 (11.42–11.9)		
**CCT**	540.27 ± 33.44	540.47 ± 33.78		**0.169**
	539 (517.5–563)	540 (518–560)		

*—significant difference.

**Table 2:** Mann–Whitney U test comparison of keratometry and astigmatism between Argos SS-OCT and Aladdin OLCI biometers (W, z, and *p*).

Argos vs. Alladin	W	z	*p*
Keratometry 1	8101	2.35	0.019 *
Keratometry 2	10,474.5	6.091	<0.001 *
Astigmatism axis (degrees)	6994	0.656	0.512
Cylinder	4892	−3.796	<0.001 *

*—significant difference.

**Table 3:** Mann–Whitney U test comparison of keratometry and astigmatism between Argos SS-OCT and Schwind Sirius (W, z, and *p*).

Argos vs. Schwind	W	z	*p*
Keratometry 1	11,442.5	7.077	<0.001 *
Keratometry 2	12,066.5	7.869	<0.001 *
Astigmatism axis (degrees)	8503.5	3.017	0.003 *
Cylinder	4002	−4.813	<0.001 *

*—significant difference.

**Table 4:** Mann–Whitney U test comparison of keratometry and astigmatism between Aladdin OLCI biometer and Schwind Sirius (W, z, and *p*).

Alladin vs. Schwind	W	z	*p*
Keratometry 1	11,162	6.436	<0.001 *
Keratometry 2	11,039.5	6.242	<0.001 *
Astigmatism axis (degrees)	8059.5	2.598	0.009 *
Cylinder	5177.5	−3.147	0.002 *

*—significant difference.

## Data Availability

The data presented in this study are available on request from the corresponding author. The data are not publicly available due to institutional data protection policies and the protection of patient confidentiality.

## Abbreviations

The following abbreviations are used in this manuscript:

D	Diopters
K	Keratometry
LT	Lens thickness
ACD	Anterior chamber depth
WTW	White-to-white
CCT	Central corneal thickness
AL	Axial length
IOL	Intraocular lens
OLCI	Optical low-coherence interferometry
SS-OCT	Swept-source optical coherence tomography
